# TGFβ-induced expression of long noncoding lincRNA Platr18 controls breast cancer axonogenesis

**DOI:** 10.26508/lsa.202101261

**Published:** 2021-11-22

**Authors:** Simon Grelet, Cécile Fréreux, Clémence Obellianne, Ken Noguchi, Breege V Howley, Annamarie C Dalton, Philip H Howe

**Affiliations:** 1 Department of Biochemistry and Molecular Biology, College of Medicine, University of South Alabama, Mobile, AL, USA; 2 Mitchell Cancer Institute, The University of South Alabama, Mobile, AL, USA; 3 Hollings Cancer Center, Medical University of South Carolina, Charleston, SC, USA; 4 Department of Biochemistry and Molecular Biology, Medical University of South Carolina, Charleston, SC, USA; 5 Department of Cell and Molecular Pharmacology and Experimental Therapeutics, Medical University of South Carolina, Charleston, SC, USA; 6 Center for Family Medicine, Sioux Falls, SD, USA

## Abstract

Metastasis is the leading driver of cancer-related death. Tumor cell plasticity associated with the epithelial–mesenchymal transition (EMT), an embryonic program also observed in carcinomas, has been proposed to explain the colonization of distant organs by the primary tumor cells. Many studies have established correlations between EMT marker expression in the primary tumor and metastasis in vivo. However, the longstanding model of EMT-transitioned cells disseminating to secondary sites is still actively debated and hybrid states are presently considered as more relevant during tumor progression and metastasis. Here, we describe an unexplored role of EMT on the tumor microenvironment by controlling tumor innervation. Using in vitro and in vivo breast tumor progression models, we demonstrate that TGFβ-mediated tumor cell EMT triggers the expression of the embryonic LincRNA Platr18 those elevated expression controls the expression of the axon guidance protein semaphorin-4F and other neuron-related molecules such as IGSF11/VSIG-3. Platr18/Sema4F axis silencing abrogates axonogenesis and attenuates metastasis. Our observations suggest that EMT-transitioned cells are also locally required in the primary tumor to support distant dissemination by promoting axonogenesis, a biological process known for its role in metastatic progression of breast cancer.

## Abstract (precis)

Tumor axonogenesis is an emerging hallmark of cancer and TGF-beta is a well-known cytokine involved in the control of cancer progression. In this study we identify a novel function for the TGF-beta signaling in cancer aggressivity by promoting cancer axonogenesis.

## Introduction

Although metastasis is the overwhelming cause of mortality in patients with solid tumors, the molecular and cellular mechanisms that drive tumor cells to become metastatic remain largely unknown (Weigelt et al, 2005; Gupta & Massagué, 2006; Chaffer & Weinberg, 2011). Epithelial-mesenchymal transition (EMT) occurs normally during early embryonic development as well as later in development and during wound healing in adults and is also reactivated during cancer progression and metastasis (Tsai et al, 2012; Tran et al, 2014; Krebs et al, 2017; Yang et al, 2020). The reverse process, known as mesenchymal–epithelial transition, also occurs frequently during health and disease. While occurring in vivo during normal development or in a pathological context, the EMT program is often incomplete, resulting in cells exhibiting diverse intermediate states that maintain both epithelial and mesenchymal characteristics depending on the biological context (Nieto et al, 2016; Jolly et al, 2019; Pastushenko & Blanpain, 2019; Yang et al, 2020). Overall, the precise role of EMT programs in tumor invasion remains highly investigated and discussed among the scientific community (Fischer et al, 2015; Ye & Weinberg, 2015; Zheng et al, 2015; Smith & Bhowmick, 2016; Aiello et al, 2017; Brabletz et al, 2018; Dongre & Weinberg, 2018).

It has been described that nerve fibers and neuron progenitors can infiltrate the primary tumor during cancer development (Mauffrey et al, 2019). The nerve density in solid tumors has also been clearly correlated to poor clinical outcomes in many cancer types including breast carcinomas (Magnon et al, 2013; Kamiya et al, 2019; Mauffrey et al, 2019). Hence, the little-understood molecular mechanisms of cancer–nerve crosstalk during cancer-associated neural infiltration represent opportunities for therapeutic intervention and needs further investigation.

Long noncoding RNAs (lncRNAs) are transcripts greater than 200 nucleotides deprived of any protein coding potential. They are divided into five broad categories, including sense, antisense, bidirectional, intronic, and intergenic, with respect to the nearest protein-coding transcripts (Meseure et al, 2015). LncRNAs modulate gene expression through different cis- or trans-acting mechanisms and they now represent essential factors in the regulation of tumor cell plasticity (Grelet et al, 2017b). Many LncRNAs are dysregulated in cancers and they have emerged as critical regulators of tumor metastasis; however, their functional integration into biological programs mediating tumor progression still needs further investigation (Bhan et al, 2017).

hnRNP E1 (PCBP1) regulates alternative splicing, mRNA stabilization, and protein translation (Grelet & Howe, 2019). The binding of hnRNP E1 to BAT structural motifs (TGFβ-activated translation) located in the 3′-UTR of a set of mesenchymal transcripts maintains epithelial cell integrity (Chaudhury et al, 2010; Hussey et al, 2011). We also identified an alternative splicing event occurring early during hnRNP E1–mediated tumor cell EMT that triggers the expression of the lncRNA PNUTS and neutralizes miR205 bioavailability in epithelial cells (Grelet et al, 2017a).

Whereas most of the mechanisms involving hnRNP E1 in tumor progression are linked to the post-transcriptional regulation of gene-expression, the impact of hnRNP E1 alteration in the transcriptional landscape of tumor cells requires further investigation. Herein, we extend our knowledge regarding the function of hnRNP E1 in the transcriptional regulation of gene expression using genome-wide unbiased approaches. By exploring the dynamics of LncRNA regulation in a hnRNP E1–mediated mouse breast cancer progression model, we identify how primary tumor cell EMT could trigger tumor axonogenesis and metastasis and validated the correlation between the EMT program and axonogenesis in the context of human breast carcinomas.

## Results

### EMT-regulated embryonic LncRNAs

**Figure 1.**
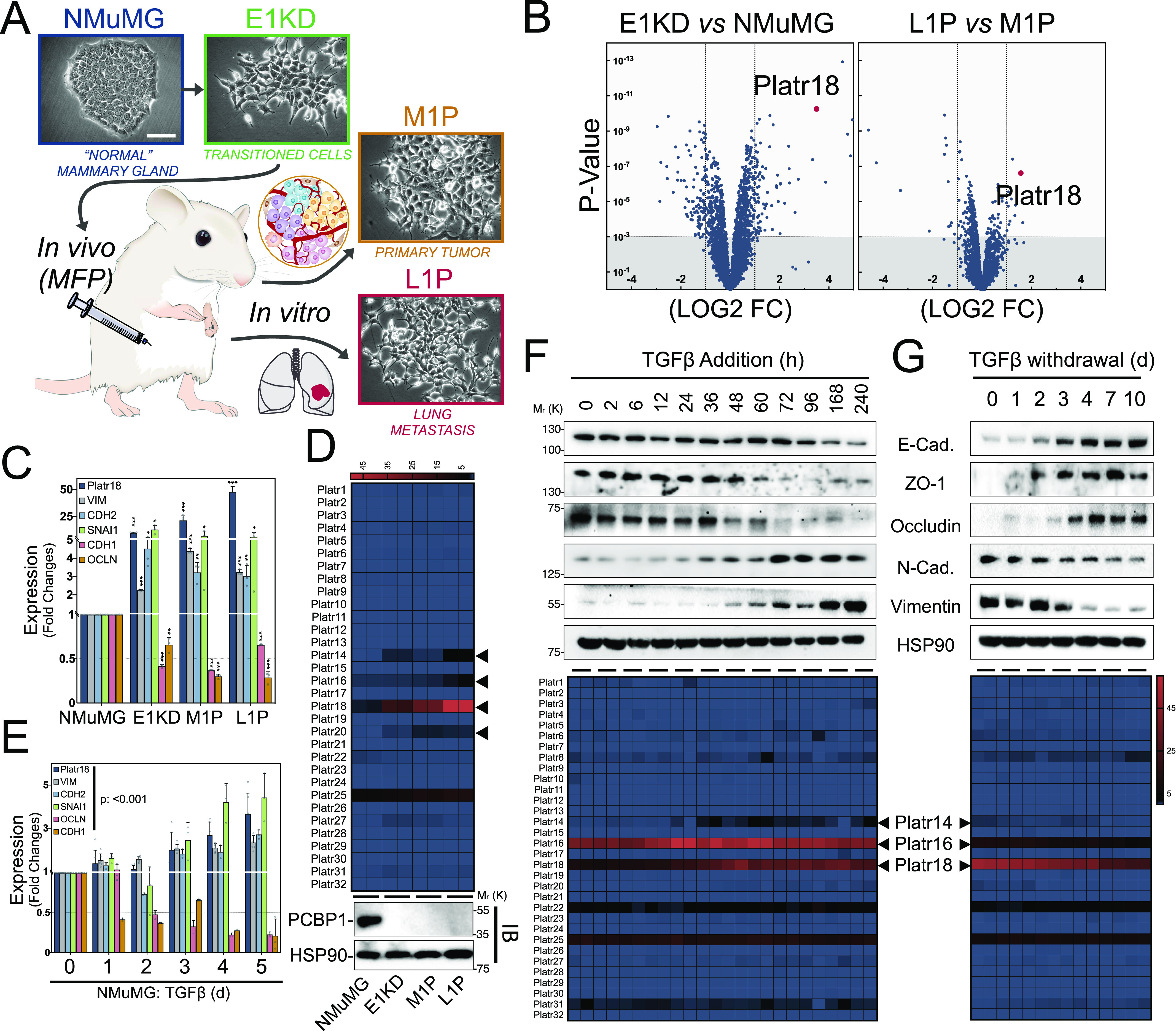
Regulation of Platr embryonic long noncoding RNAs during epithelial–mesenchymal transition (EMT). **(A)** Schematic of the mouse tumor progression model. Non-transformed normal murine mammary gland (NMuMG) cells were silenced for hnRNP E1 and injected into the mammary fat pad of 6-wk old NOD/SCID mice. After ∼12 wk, cells from the primary tumor (M1P) and lung metastases (L1P) were isolated and harvested for culture in vitro. Each established cell line is presented on phase contrast micrographs. **(B)** Volcano plots of the RNA-Seq analysis of noncoding transcripts expression at the EMT (*E1KD* versus *NMuMG*) and metastatic (*L1P* versus *M1P*) steps of tumor progression. Only long noncoding transcripts are plotted. **(C)** Quantitative RT-PCR analysis of Platr18 transcript, mesenchymal markers vimentin (VIM), N-Cadherin (CDH2) and snail (SNAI1), epithelial markers occludin (OCLN) and E-cadherin (CDH1) in hnRNP E1–derived cells. Data are normalized to HPRT and expressed in fold changes compared with the control with mean ± SD. ANOVA *P* < 0.0001. **(D)** (Top) Heat map from RNA-Seq analysis of Platrs expression in the NMuMG-derived breast tumor progression model. Each cell line is in duplicate. (Bottom) Immunoblot analysis of PCBP1 expression in the tumor progression series. **(E)** Quantitative RT-PCR analysis of Platr18 transcript, mesenchymal markers vimentin (VIM), N-Cadherin (CDH2) and Snail (SNAI1), epithelial markers occludin (OCLN) and E-cadherin (CDH1) transcripts expression during TGFβ-mediated EMT in NMuMG cells. Data are normalized to HPRT and expressed in fold changes compared to the control with mean ± SD (n = 3–6). **(F, G)** Immunoblot analysis (top panel) of E-cadherin, ZO1 and occludin epithelial markers and vimentin and N-cadherin mesenchymal markers expression during TGFβ-induced EMT (F) or TGFβ-retrieval model of mesenchymal–epithelial transition after 10 d of continuous TGFβ exposure in NMuMG cells (G). Heat map (bottom panel) of Platr noncoding RNA genes expression during TGFβ-mediated cell EMT of NMuMG cells. RNA-Seq data extracted from GSE112797 with each time point in duplicate (n = 2). Data are mean ± SD; NS, nonsignificant, **P < 0.05, ***P < 0.01, ***P < 0.001, or *P*-values are from ANOVA. HSP90 serve as loading controls. Experiments have been repeated three times or as specified in the legend. Scale bars: 50 μm.

**Figure S1.**
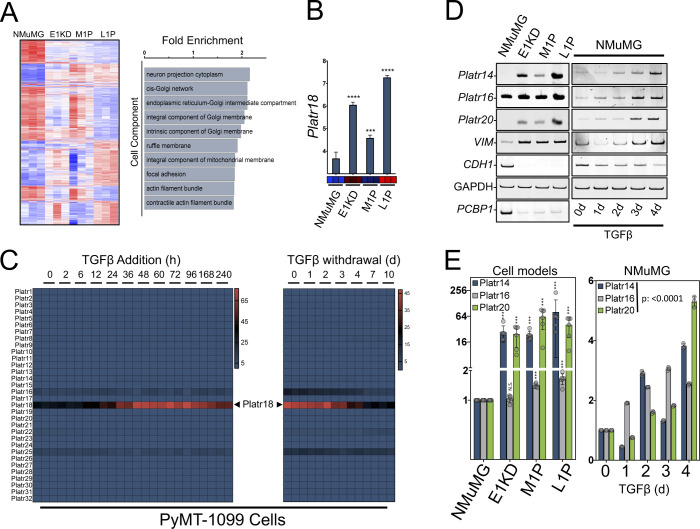
Platr non-coding genes are reactivated during cancer cell plasticity and metastasis. **(A)** Affymetrix transcriptomic analysis of genes expression in the tumor progression model. Heat map showing the differentially expressed genes across the series. Affymetrix array from GSE94637 with each sample in triplicate (left). Gene ontology term enrichment classification by cell component of the set of the most regulated genes (F score >5) (right). **(B)** Affymetrix expression profile of Platr18 in the NMuMG-derived breast tumor progression model. **(C)** Heat map of Platrs expression in the TGFβ-induced epithelial–mesenchymal transition (left) or TGFβ-retrieval model of mesenchymal–epithelial transition after 10 d of continuous TGFβ exposure (right) of the PyMT-1099 cell line derived from mouse mammary tumor virus-PyVT model of breast cancer. RNA-seq data extracted from (GSE114572) with each time point in duplicate. **(D, E)** Platr-14/-16/-20 expression in NMuMG-derived breast tumor progression and TGFβ-mediated epithelial–mesenchymal transition analysis by using (D) end point PCR and (E) quantitative PCR. (n = 6) Data are mean ± SD; NS, nonsignificant; ***P < 0.001 or *P*-value from ANOVA. GAPDH serves as a loading control. Experiments have been repeated three times or as specified in the legend.

We and others previously showed that TGFβ-mediated phosphorylation of hnRNP E1/PCBP1 or its silencing induces EMT (Chaudhury et al, 2010; Song et al, 2014; Grelet et al, 2017a; Grelet & Howe, 2019). This switch mimics TGFβ exposure of normal epithelial or carcinoma cells and activates the tumorigenicity of normal murine mammary gland (NMuMG) epithelial cells (Chaudhury et al, 2010; Hussey et al, 2011). We developed a mouse model of breast tumor progression by orthotopic injection of NMuMG cells silenced for hnRNP E1 (E1KD) (Fig 1A) (Howley et al, 2017) and collected primary tumor (M1P) and distant metastasis (L1P) cells to study LncRNAs regulation. High throughput RNA-sequencing (RNA-Seq) analysis identified 186 & 26 noncoding transcripts differentially expressed in “EMT” (*E1KD* versus *NMUMG*) or “metastasis” (*L1P* versus *M1P*) steps of tumor progression, respectively (Fig 1B). *Platr18* long intergenic non–protein-coding RNA (LincRNA), also known as *LncEnc1*, was the most commonly regulated transcript as well as the highest hit during metastasis. *Platr18* dynamics were validated by RT-PCR (Fig 1C) and mining from a previous microarray experiment (Howley et al, 2017) ranked it at the 99.95 percentile of the entire dataset and as the most regulated LncRNA across the series (Fig S1A and B and Table S1). TGFβ triggers a strong EMT and dynamically up-regulates Platr18 expression that is reversed upon TGFβ withdrawal in both NMuMG cells (Fig 1E–G) and in the PyMT-1099 cell line established by the Christofori’s group (Saxena et al, 2018) (Fig S1C).

Table S1 Most regulated transcripts in the Affymetrix array dataset (GSE94637).

*Platr18* is the 18^th^ member of the Platr noncoding RNA cluster (pluripotency-associated transcript) required in the maintenance of embryonic stem cells (ESCs) (Ivanova et al, 2006; Guttman et al, 2011; Sun et al, 2018). Platr transcripts do not share commonalities other than their tight association with the pluripotent state of ESCs but other members (Platr14, 16, and 20) are also significantly reactivated during both tumor progression and TGFβ-mediated EMT of NMuMG cells (Figs 1D, F, and G and S1D and E).

### Platr18 controls Sema4F expression

**Figure 2.**
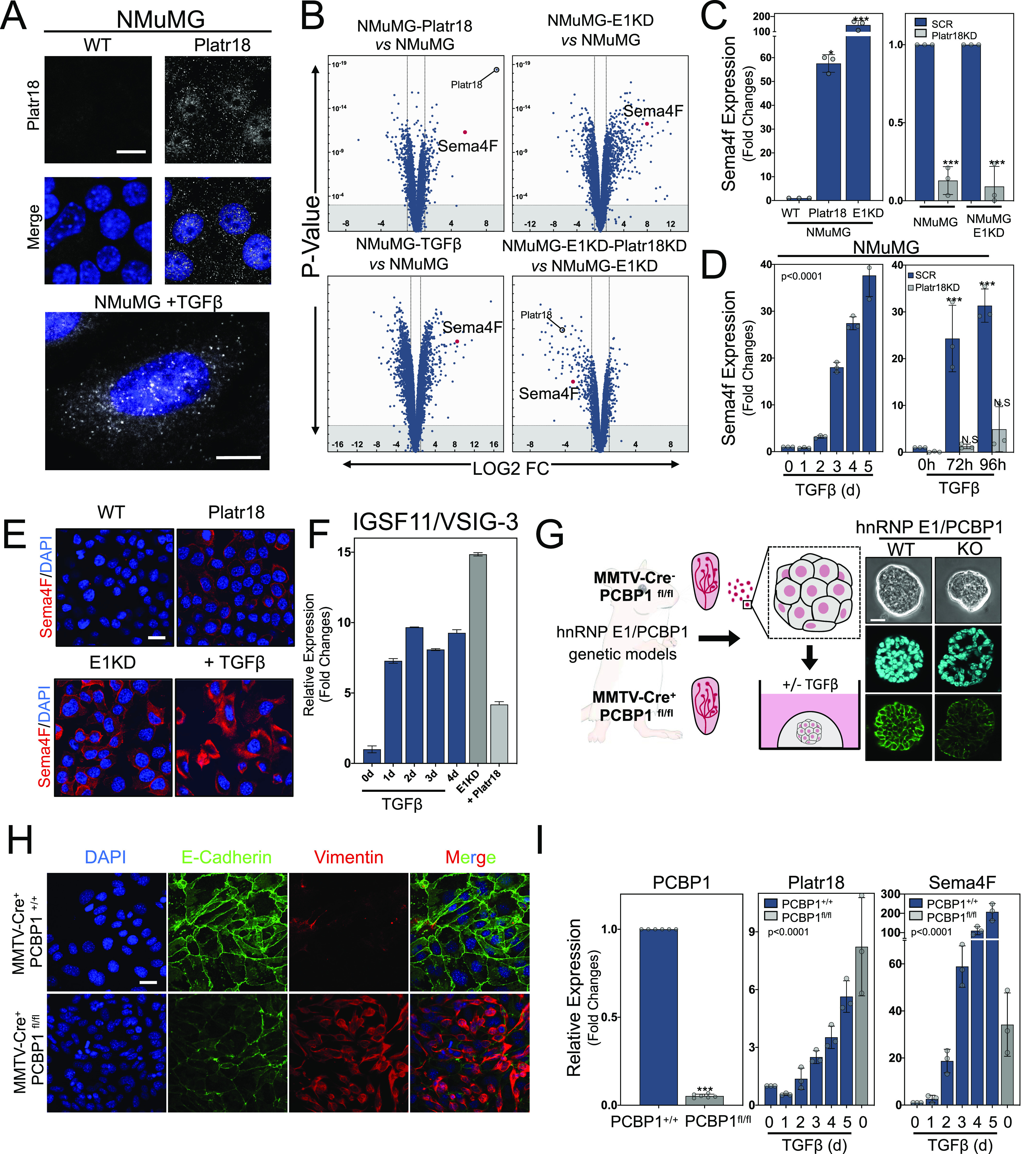
The TGFβ/E1KD/Platr18 axis controls semaphorin-4F expression in vitro and in vivo. **(A)** Single-molecule RNA-FISH labeling of NMuMG cells overexpressing Platr18 LincRNA or treated with TGFβ (bottom). Scale bar: 10 μm. **(B)** Volcano plot of the genome-wide transcriptome analysis of NMuMG overexpressing Platr18 (NMuMG-Platr18), silenced for hnRNP E1 (NMuMG-E1KD), treated with TGFβ for 4 d (NMuMG-TGFβ), and silenced for Platr18 in NMuMG-E1KD (NMuMG-E1KD-Platr18 KD). All genes are plotted. **(C)** (Left) Sema4F transcript expression in NMUMG cells overexpressing (+Platr18) or silenced for hnRNP E1(E1KD). (Right) Sema4F transcript expression after Platr18 silencing (Platr18 KD) in NMuMG or NMuMG silenced for hnRNP-E1 expression (NMuMG E1KD). Data are expressed in fold changes compared to the control with mean ± SD; *P < 0.05; ***P < 0.001 (n = 3). **(D)** Time course of Sema4F transcript expression under TGFβ exposure in (left) NMuMG cells or (right) NMuMG cells silenced for Platr18 expression (SCR versus Platr18 KD cells). Data are expressed in fold changes compared to the control with mean ± SD. TGFβ treatment: ANOVA *P* < 0.0001; ***P < 0.001; NS, not significant (n = 3). **(E)** Immunofluorescence of Sema4F protein expression and localization in NMUMG cells overexpressing Platr18 (NMUMG-Platr18), silenced for E1KD (NMUMG-E1KD), or treated with TGFβ (NMUMG + TGFβ) for 3 d. **(F)** Time course of IGSF11/VSIG3 transcript expression under TGFβ exposure, hnRNP E1 silencing (E1KD), or Platr18 expression. (n = 3) Scale bar: 20 μm. **(G)** Organoid cultures derived from mouse mammary tumor virus (MMTV)-Cre^−^ PCBP1^fl/fl^ or MMTV-Cre^+^ PCBP1^fl/fl^. Phase-contrast micrograph of organoid morphology and E-cadherin immunofluorescence (green) with nuclei counterstained by DAPI (blue). 60X magnification. Scale bar: 20 μm. **(H)** Immunofluorescence analysis of E-Cadherin and vimentin markers expression in MMTV-Cre^−^ PCBP1^fl/fl^ or MMTV-Cre^+^ PCBP1^fl/fl^ hTert-immortalized cells in culture in vitro. Scale bar: 20 μm. **(I)** Quantitative PCR analysis of PCBP1 (hnRNP E1), Platr18, and Sema4F expression in 2D cultures derived from MMTV-Cre^−^ PCBP1^fl/fl^ and MMTV-Cre^+^ PCBP1^fl/fl^ mice after exposure to TGFβ (n = 3). Data are expressed in fold changes compared to the control with mean ± SD; ***P < 0.001; ANOVA *P* < 0.0001. Experiments have been repeated three times or as specified in the legend.

**Figure S2.**
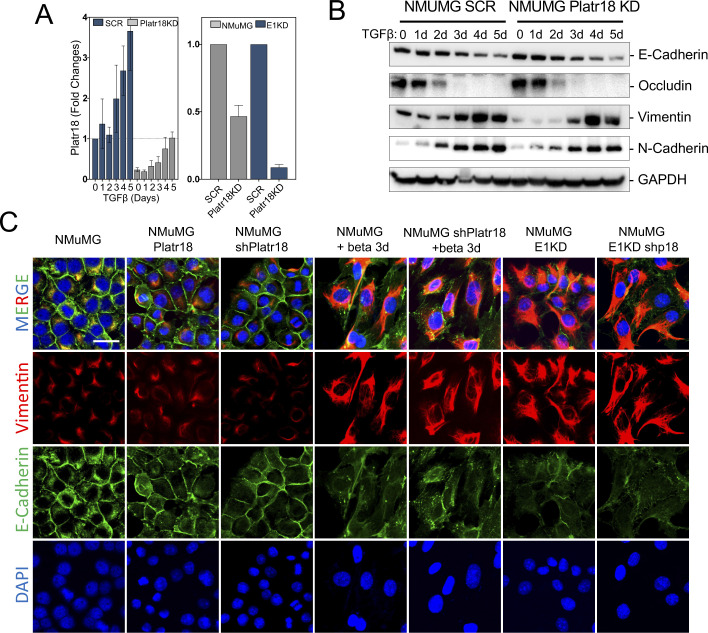
Platr18 silencing. **(A)** Characterization of Platr18 silencing. Quantitative PCR analysis of Platr18 relative expression in NMuMG cells expressing scrambled (SCR) or Platr18 specific (Platr18 KD) shRNAs and treated with TGFβ (0–5 d; 5 ng.mL^−1^) (left) or silenced for hnRNP E1 (E1KD) (right). (n = 3) Data are mean ± SD. **(B)** Platr18 lincRNA silencing does not prevent TGFβ-induced epithelial–mesenchymal transition. Western-blot analysis of epithelial (E-cadherin; occludin) and mesenchymal (vimentin; N-cadherin) markers expression in NMuMG cells expressing scrambled (SCR) or Platr18 specific (Platr18 KD) shRNAs and treated with TGFβ for up to 5 d. **(C)** Immunofluorescence staining of vimentin/E-cadherin expression in NMuMG cells overexpressing Platr18 (Platr18), silenced for Platr18 (shPlatr18), and treated TGFβ (+beta 3 d) or after silencing of hnRNP E1 (E1KD). Experiments have been repeated three times or as specified in the legend. Scale bar: 20 μm.

**Figure S3.**
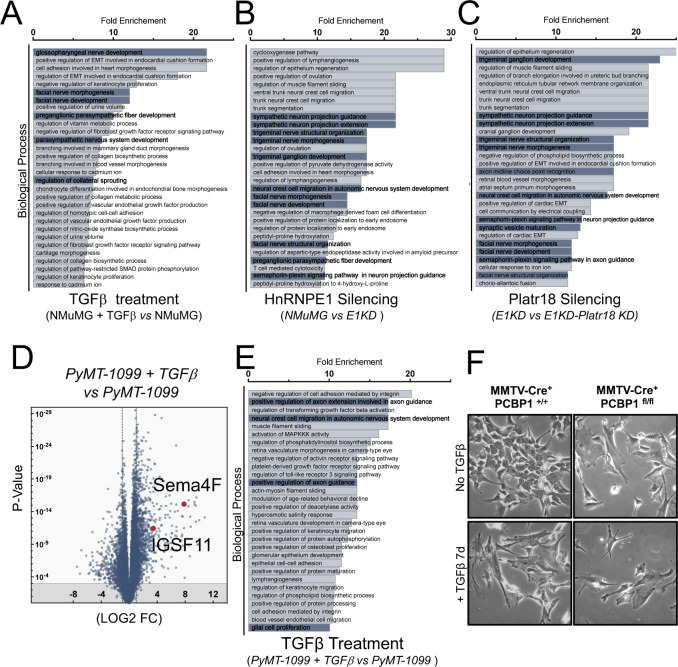
TGFβ/hnRNPE1/Platr18 axis controls neurogenesis-related genes expression. **(A, B, C)** RNA-seq experiment interpretation by gene ontology term enrichment classification (biological process) of the transcripts up-regulated in NMuMG cells treated with TGFβ (A) up-regulated in NMuMG cells silenced for hnRNP E1 (B) or down-regulated in E1KD cells silenced for Platr18 expression (C). **(D)** Sema4F is highly regulated gene during TGFβ-mediated epithelial–mesenchymal transition of PyMT-derived cells. RNA-Seq analysis of the transcriptomes of PyMT-1099 cells treated with TGFβ for 96 h (*PyMT-1099 + TGF*β versus *PyMT-1099*). Data extracted from (GSE114572). All genes are plotted. **(E)** Gene ontology term enrichment classification of up-regulated transcripts in PyMT-1099 cells treated with TGFβ. **(F)** Phase contrast micrograph of 2D cultures of mouse mammary tumor virus-Cre^−^ PCBP1^fl/fl^ and mouse mammary tumor virus-Cre^+^PCBP1 ^fl/fl^ ± TGFβ treatment for 7 d.

Platr18 is among the more robustly regulated transcripts in the tumor progression series and is the most regulated noncoding transcript after hnRNP E1 silencing. Single-molecule RNA-FISH revealed its nuclear enrichment after its forced overexpression or its up-regulation by TGFβ treatment of the NMuMG cells (Fig 2A), suggesting a functional integration into the transcriptional program of EMT as it was observed in the context of ESCs (Bergmann et al, 2015; Sun et al, 2018). Transcriptome signature analysis of cells modulated for Platr18, hnRNP E1, or treated with TGFβ (Fig 2B) revealed semaphorin-4F (Sema4F) as the most regulated transcript by Platr18 overexpression itself (Figs 2B and C and S2A) and one of the most modulated RNAs during tumor cell EMT in general (Fig 2B and D). In NMuMG cells, Sema4F expression is up-regulated by ∼60 and ∼140-fold after Platr18 overexpression or hnRNP E1 silencing, respectively (Fig 2C). Although Platr18 silencing does not prevent TGFβ-induced EMT (Fig S2B and C), it abolishes both hnRNP E1-silencing and TGFβ-dependent up-regulation of Sema4F in NMuMG cells in vitro (Fig 2C and D). Sema4F dynamics were further validated at the protein level by immunofluorescence in NMuMG cells (Fig 2E) and also, in the PyMT-1099 model where the transcript was induced by ∼230-fold after TGFβ treatment (Fig S3D).

We developed a floxed transgenic mouse model of mammary specific hnRNP E1/PCBP1 knockout (Ryu et al, 2017) upon crossing with a mouse mammary tumor virus (MMTV) promoter driven Cre recombinase mouse (Fig 2G). Both MMTV-Cre^−^/PCBP1^fl/fl^ and MMTV-Cre^+^/PCBP1^fl/fl^ mammary glands were processed to generate 2D or organoids cultures (Figs 2G and S3F). Quantitative analysis validated a ∼95% decrease in hnRNP E1 transcript in the MMTV-Cre^+^/PCBP1^fl/fl^ organoids (Fig 2I) and these display a disorganized morphology associated with decreased expression of E-cadherin (Fig 2G) and the primary cells immortalized by hTERT exhibit an EMT^+^ phenotype in 2D culture in vitro, whereas deleted for hnRNP E1 (Fig 2H). In MMTV-Cre^−^/PCBP1^fl/fl^ organoid–derived cells, TGFβ induced Platr18 and Sema4F in a time-dependent manner with maximal effects of ∼6-fold and ∼200-fold observed after a 5-d treatment, respectively, whereas in MMTV-Cre^+^/PCBP1^fl/fl^ organoids, mere deletion of hnRNP E1 was sufficient to induce Platr18 (∼8-fold) and Sema4F (∼35-fold) (Fig 2I).

### Sema4F controls tumor axonogenesis

**Figure S4.**
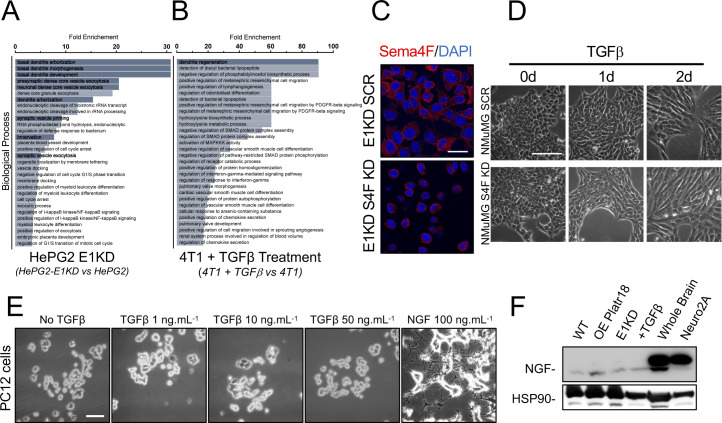
Supplementary figure 4. **(A)** Gene ontology term enrichment classification of transcripts up-regulated in human liver carcinoma HePG2 cells silenced for hnRNP E1. RNA-Seq data extracted from the ENCODE project ENCSR603TCV & ENCSR635FRH. **(B)** Gene ontology term enrichment classification of transcripts up-regulated in 4T1 cells treated with TGFβ for 24 h. RNA-seq data extracted from GSE110912. **(C)** Validation of Sema4F silencing. Immunofluorescence staining of E1KD cells expressing scrambled (E1KD SCR) or Sema4F-specific shRNAs (E1KD S4F KD). Nuclei are counterstained with DAPI. Scale bar: 20 μm. **(D)** Sema4F silencing does not impair TGFβ-mediated changes in cell morphology. Phase contrast micrographs of NMuMG cells expressing scrambled (NMuMG SCR) or Sema4F specific (NMuMG S4F KD) shRNAs and treated with TGFβ for up to 2 d (0–2 d; 5 ng.mL^−1^). Scale bar: 100 μm. **(E)** PC12 cells do not differentiate after TGFβ exposure. Bright-field micrographs of PC12 cultures exposed to TGFβ (1–50 ng.mL^−1^) for 5 d. Nerve growth factor (NGF) serves as a positive control. Scale bar: 50 μm. **(F)** NMuMG-derived models do not produce significant amount of NGF. Western blot analysis of NGF production from whole cell lysates of NMuMG cells (WT), NMuMG cells overexpressing Platr18 (OE Platr18), silenced for hnRNP E1 (E1KD), or treated with TGFβ for 3 d. Mouse whole brain (Whole Brain) and N2A cell line (Neuro2A) lysates are used as positive controls. HSP90 is used as a loading control. Experiments have been repeated three times or as specified in the legend.

Gene ontology analysis of RNA-Seq and Affymetrix array experiments in NMuMG and PyMT-1099 models identified a systematic enrichment of processes involved in neuronal development (Figs S1A and S3A–E). Beyond Sema4F, multiple axon-related molecules, classified for their function in tumor axonogenesis, such as Bmp7, Robo1, Fibronectin, or Nrp1, and the synaptic adhesion molecule IGSF11/VSIG-3 are regulated upon TGFβ−mediated EMT induction, hnRNP E1 silencing, or Platr18 overexpression (Fig 2F and Tables S2 and S3). Furthermore, RNA-seq analysis of human hepatocellular carcinoma (HepG2) cells silenced for hnRNP E1 (Fig S4A) and from murine 4T1 breast cancer cells treated with TGFβ (Fig S4B) also showed neuron-related factor enrichment, although these models do not display regulation for Platr18/Sema4F. Overall, we observed a link between TGFβ-mediated EMT and tumor axonogenesis; however, by crossing the various models, no consensus emerged about a canonical axis involved in the TGFβ-mediated tumor axonogenesis. This observation suggests that EMT may act as a trigger for tumor axonogenesis in a more general manner and that the E1KD/Platr18/Sema4F axis is replaced according to the specific biological context.

Table S2 Axon-related factors regulated upon Platr18 overexpression in NMuMG cells.

Table S3 Axon-related factors regulated upon Platr18 overexpression in NMuMG cells.

**Figure 3.**
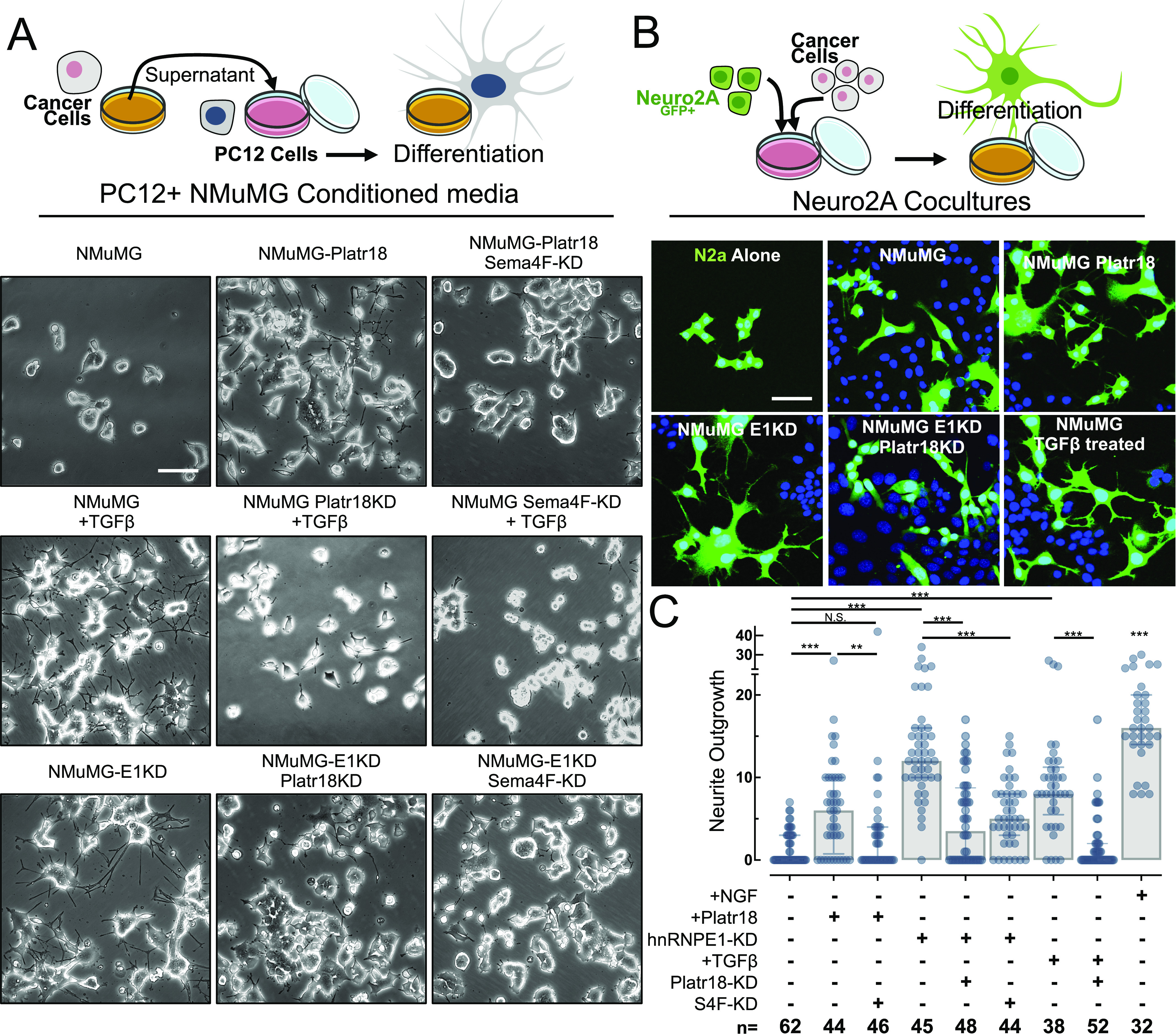
The TGFβ/E1KD/Platr18 axis controls axonogenesis in vitro. **(A)** Schematic of the PC12 axonogenesis assay in vitro (top panel). PC12 cells derived from pheochromocytoma of the rat adrenal medulla are exposed to culture supernatants of tumor cells (lower panels). Micrographs of PC12 cells complemented with cultured media from NMuMG cells (*NMuMG*), overexpressing Platr18 (*-Platr18*) silenced for Sema4F expression (*Sema4F-KD*), silenced for hnRNP E1 (*-E1KD*), silenced for Platr18 (Platr18 KD) and treated with TGFβ (+*TGF*β). **(B)** Schematic of the Neuro2A axonogenesis co-culture assay in vitro (top panel). Mouse Neuro2A neuroblastoma cells are transduced with a lentivirus carrying the eGFP gene, FACS-sorted, and then cultured in the presence of tumor cells. The neurite outgrowth is tracked by fluorescence microscopy that allows distinguishing the neuronal component of the co-culture through the GFP expression (bottom panels). *DAPI*^*+*^*/eGFP*^*+*^ = Neuro2A; *DAPI*^*+*^*/eGFP*^*−*^ = tumor cells. Neuro2A confocal microscopy pictures of the Neuro-2A cells cultivated alone (N2a Alone) or in the presence of NMuMG cells overexpressing Platr18 (*Platr18*), silenced for hnRNP E1 (*E1KD*), silenced for Platr18 (*Platr18 KD*), or pretreated with TGFβ prior (not during the co-culture) their addition to the Neuro2A^GFP+^ culture. **(C)** Scatter plot of the neurite outgrowth absolute quantification of the PC12 axonogenesis assay in vitro. For each culture condition (bottom table) the number of dendrites per cell is reported. (n=) number of PC12 cells per condition. The histogram displays the median ± interquartile range. *P < 0.05; **P < 0.01; ***P < 0.001. Experiments have been repeated three times or as specified in the legend. Scale bars: 50 μm.

To validate the pro-axonogenesis role of EMT, we used two axonogenesis models in vitro (Fig 3). In pheochromocytoma (PC12) cells (Fig 3A), addition of wild-type NMuMG supernatants had little effect on neuronal differentiation, whereas Plat18 overexpression, hnRNP E1 silencing, or TGFβ treatment of NMuMG cells produced cellular supernatants that trigger PC12 cell axonogenesis characterized by neurites sprouting (Fig 3A). Although Platr18 (NMuMG Platr18 KD) or Sema4F (NMuMG Sema4F-KD) silencing does not prevent TGFβ-mediated EMT of NMuMG (Figs S2B and S4C and D), it significantly blocks neuronal differentiation of PC12 induced by Platr18, E1KD, and TGFβ-treated NMuMG supernatants (Fig 3A and C).

The role of Platr18/Sema4F in EMT-induced axonogenesis was further validated in the mouse neuroblastoma Neuro2A^GTP+^ coculture model (Fig 3B). As observed by fluorescence microscopy, Neuro2A^GTP+^ cells are more differentiated when co-cultured with either NMUMG cells overexpressing Platr18, silenced for hnRNP E1, or pretreated with TGFβ than wild-type NMuMG, and this effect is reduced under Platr18 silencing (Fig 3B).

### Platr18/Sema4F and tumor axonogenesis in vivo

**Figure 4.**
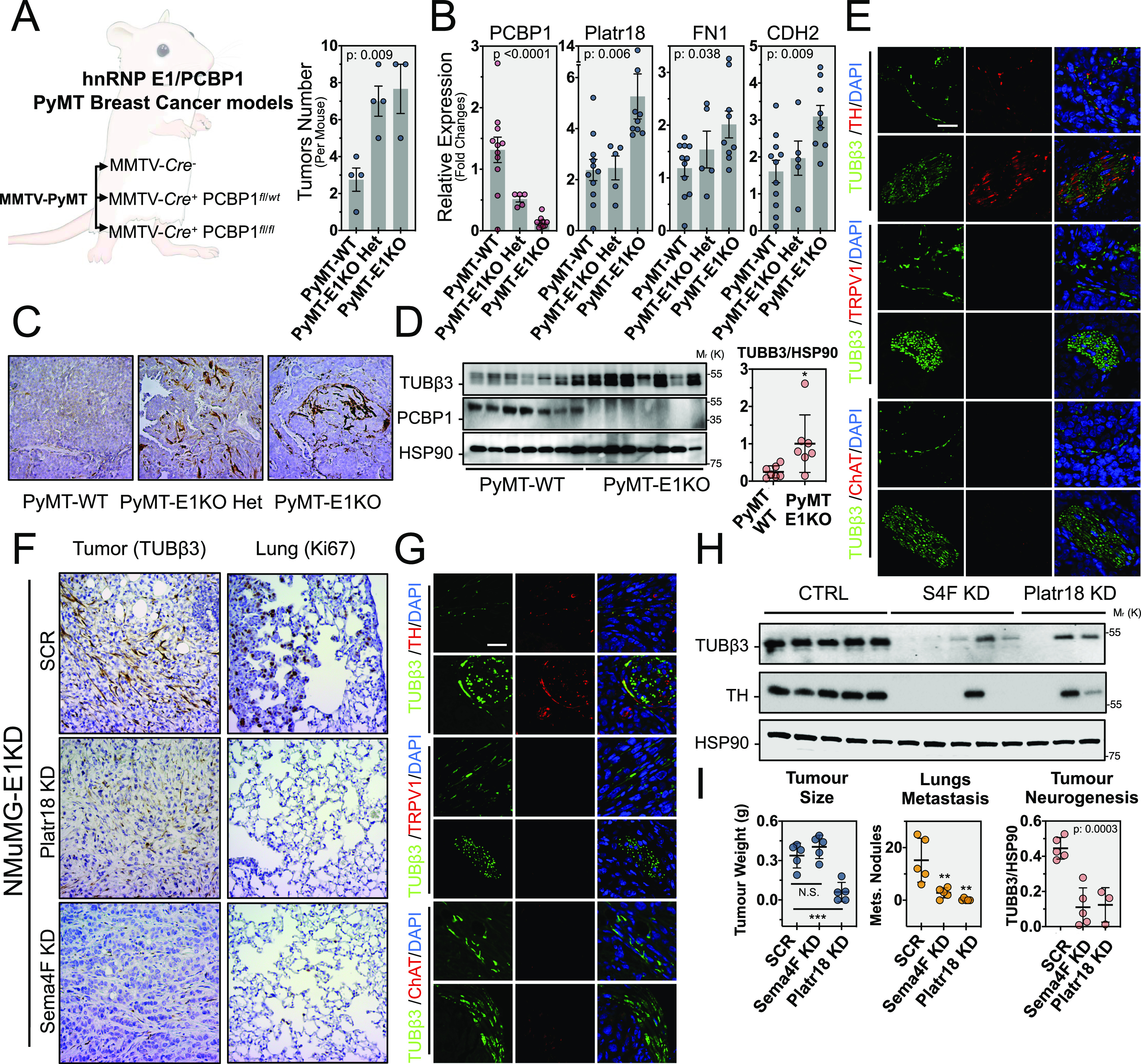
E1KD/Platr18/Sema4F axis controls primary tumor axonogenesis in vivo. **(A)** hNRNP E1/PCBP1 genetic deletion in mouse mammary tumor virus (MMTV)/PyMT-driven breast cancer progression model in vivo. MMTV-PyMT, MMTV-Cre^−^ (PyMT-WT), MMTV-PyMT, MMTV-Cre^+^ PCBP1^fl/wt^ (PyMT-E1KO-Het) and MMTV-PyMT, MMTV-Cre^+^ PCBP1^fl/fl^ (PyMT-E1KO), mouse genetic models were generated. Histograms represent the number of tumors observed per mouse for each group. Data expressed as mean ± SEM. *P*-value from ANOVA (n = 4). **(B)** Quantitative RT-PCR analysis of hnRNP E1 (PCBP1), Platr18, fibronectin (FN1), and N-cadherin (CDH2) expression in the primary tumors. Data are normalized to HPRT and expressed in fold changes compared with the control with mean ± SEM. *P*-values from ANOVA. (n = 5–11 as indicated). **(C)** Tubulin β3 (Tubβ3) IHC staining on primary tumors. 20× magnification. **(D)** (Left) Western blot analysis of neuron-specific tubulin β3 (Tubβ3) marker and hnRNP E1/PCBP1 expression in primary tumors of PyMT-WT and PyMT-E1KO primary tumors and (Right) quantification of the tubulin β3 signal compared with HSP90. Mean ± SD *P < 0.05. **(E)** Characterization of the PyMT-E1KO primary tumors innervation. Co-immunofluorescence analysis of neuron-specific marker tubulin β3 (Tubβ3–green) with sympathetic-specific markers (red) tyrosine hydroxylase (TH), sensory-specific marker capsaicin receptor (TRPV1), or parasympathetic marker choline acetyltransferase (ChAT). For each marker, a representative nerve twigs or a more organized fiber is displayed. 120× magnification. **(F)** In vivo analysis of primary tumor-related axonogenesis and lung metastasis in female NOD/SCID mice injected into the mammary fat-pad with NMuMG-E1KD control cells (SCR), silenced for Platr18 (Platr18 KD), or Sema4F (Sema4F KD). Tubulin β3 (Tubβ3) and Ki67 markers are used to IHC stain tumor innervation and lung metastasis, respectively. 20× magnification. **(G)** Characterization of the primary tumors innervation in the orthotopic model. Co-immunofluorescence analysis of neuron-specific marker tubulin β3 (Tubβ3 – green) with sympathetic-specific marker tyrosine hydroxylase (TH), sensory-specific marker capsaicin receptor (TRPV1), or parasympathetic marker choline acetyltransferase (ChAT). For each marker, a representative nerve twig or a more organized fiber is displayed. 120× magnification. **(H)** Western-blot analysis of neuron-specific tubulin β3 (Tubβ3) and sympathetic-specific tyrosine hydroxylase (TH) markers expression in primary tumors. **(I)** Quantification of tumor weight (g), lung metastases (number of nodules), and tumor axonogenesis (quantification of Tubβ3 marker expression in primary tumors) of mice injected with NMuMG-E1KD control cells (SCR) or silenced for Platr18 (Platr18 KD) or Sema4F (Sema4F KD). (n = 5 mice per group) Data are mean ± SD; **P < 0.01; ***P < 0.001, or *P*-value from ANOVA. HSP90 serves as a loading control. Experiments have been repeated three times or as specified in the legend. Scale bars: 50 μm.

To elucidate the role of hnRNP E1 in the primary tumor axonogenesis, we generated a PyMT-MMTV mouse model deleted for hnRNP E1/PCBP1 expression (Fig 4). While all mice developed tumors, we observed a significant increase in their numbers after deletion of hnRNP E1 (Fig 4A). Furthermore, we validated an increase in Platr18 expression that is also correlated with increased levels of the mesenchymal markers fibronectin and N-cadherin (Fig 4B). Pathological analysis and biochemical quantification revealed that primary tumors were more innervated after partial or total deletion of hnRNP E1 (Fig 4C and D). Overall, we observed primary tumor axonogenesis consisting of nerve twigs heterogeneously distributed throughout the primary tumor and few more organized nerves fibers distributed on the tumor periphery. The specific nature of the nerve compartment was then characterized by immunofluorescence using tubulin β3 nerve-specific marker combined with tyrosine hydroxylase (sympathetic innervation), TRPV1 (capsaicin receptor; sensory innervation), or choline acetyltransferase (parasympathetic) markers. Both nerve twigs and organized fibers revealed a sympathetic-type innervation, whereas sensory or parasympathetic markers expression was not detected (Fig 4E).

Because only few metastases were observed in the PyMT-MMTV-E1 progression model, we were unable to correlate the observed changes in sympathetic innervation to distant colonization. We thus used an orthotopic mammary fat pad (MFP) injection model of NMuMG-E1KD cells that has been described for its high metastatic potential (Chaudhury et al, 2010; Hussey et al, 2011; Howley et al, 2017) (Fig 4F–I). These cells develop primary tumors embedding few organized fibers but numerous disorganized nerve twigs of sympathetic nature (Fig 4F–H) whose numbers are considerably reduced by the silencing of Platr18 or Sema4F as observed by IHC and quantified by western-blot (Fig 4H and I). Axonogenesis is linked to tumor progression and contributes to breast cancer metastasis (Zhao et al, 2014; Kuol et al, 2018; Kamiya et al, 2019) and we observed that both silencing of Platr18 and Sema4F abrogate metastasis to the lungs (Fig 4I). Interestingly, whereas Sema4-F silencing had no impact on tumor weight, Platr18-silenced tumors were smaller and only three of the five MFP injections led to detectable tumor outgrowth (Fig 4I).

### EMT-mediated tumor axonogenesis in human context

**Figure 5.**
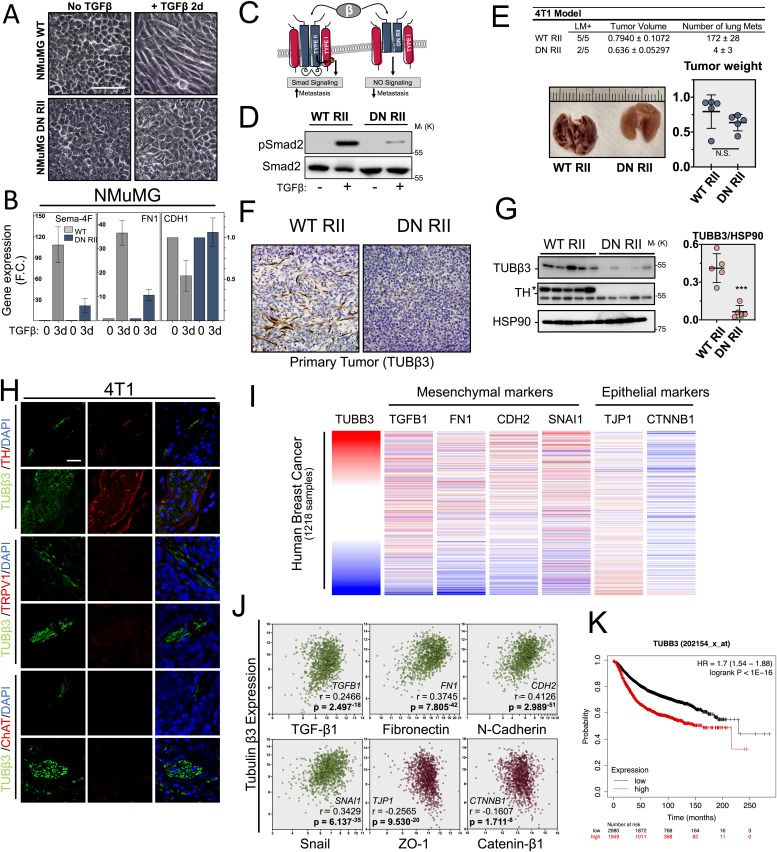
Epithelial–mesenchymal transition (EMT) controls tumor axonogenesis and metastasis in vivo. **(A)** Characterization of dominant negative TGFβ type II receptor strategy in NMuMG model. Micrographs of NMuMG cell cultures expressing WT (NMuMG WT) or dominant negative TGFβ type II receptor (NMuMG DN RII) and treated with TGFβ to induce EMT. **(B)** Quantitative PCR analysis of Sema4F, fibronectin (FN1; mesenchymal marker), and E-cadherin (CDH1; epithelial marker) transcripts induction by TGFβ in the dominant negative TGFβ type II receptor model. Data are mean ± SD (n = 3). **(C)** Schematic of the dominant negative TGFβ type II receptor (RII) expression strategy to block TGFβ signaling. **(D)** Western blot analysis of Smad2 phosphorylation 30 m after TGFβ exposure (0.5 ng.mL^−1^) in the mouse 4T1 breast cancer cells expressing either the wild-type (WT) or dominant negative TGFβ type II receptor (DN RII) in vitro. **(E)** (Top) Lung metastases quantification are reported in the table. LM+ = number of mice positive for lung metastases; five mice per group. (Bottom) Representative picture of mice lungs 3 wk after mammary fat pad injection of 4T1 cells expressing WT (WT RII) or truncated TGFβ type II receptor (DN RII) & obtained tumors weight (g). Data are mean ± SD (n = 5 mice per group). **(F)** Tumor-related axonogenesis of tumors generated by mammary fat pad injection of NOD/SCID mice with 4T1 cells enabled (WT) or blocked (DN RII) for TGFβ signaling. Tubulin β3 immunostaining visualizes intra-tumoral innervation. **(G)** Western blot analysis of axonogenesis in tumors abolished for TGFβ signaling. Tubulin β3 and TH neuronal markers were used to quantify axonogenesis. Densitometry quantification of Tubulin β3 is provided in scattered dot plots on the right of the blots. Data are mean ± SD; ***P < 0.001. HSP90 is used as a loading control. (n = 5 mice per group). **(H)** Characterization of primary tumors innervation in the 4T1 orthotopic model of tumor progression. Co-immunofluorescence analysis of neuron-specific marker Tubulin β3 (Tubβ3 – green) with sympathetic-specific marker tyrosine hydroxylase (TH), sensory-specific marker capsaicin receptor (TRPV1), or parasympathetic marker choline acetyltransferase (ChAT). For each marker, a representative nerve twig or a more organized fiber is displayed. 120× magnification. **(I)** Cross-correlation between EMT and tumor innervation in human breast cancer samples. (Top) Analysis of human breast cancer primary tumor axonogenesis (TUBB3 gene expression) and EMT-related mesenchymal (TGFβ1, FN1, CDH2, and SNAI1) and epithelial (TJP1 and CTNNB1) genes expression in 1,218 human breast tumor samples from the Cancer Genome Atlas BRCA RNA-sequencing database. Tumor innervation is ranked through tubulin-β3 expression analysis. Red color indicates tumors with the highest innervation. **(J)** Correlation analysis between primary tumor axonogenesis and EMT related factors. Genes expression is expressed in Log_2_ (normalized count + 1) and Pearson’s rho (r=) and *P*-value (p=) is provided for each correlation. HSP90 serves as a loading control. **(K)** Kaplan–Meier survival curve comparing breast cancer innervation to overall survival. Data obtained from the KM plotter breast dataset in overall breast cancer samples (n = 4,929). HSP90 serves as a loading control. Experiments have been repeated three times or as specified in the legend. Scale bars: 50 μm.

Finally, we extended our investigations to tumor cell EMT as a whole by blocking TGFβ signaling using a dominant negative strategy (Fig 5A–H) (Herskowitz, 1987; Chen et al, 1993; Portella et al, 1998; Tang et al, 1999; McEarchern et al, 2001). Expression of dominant negative TGFβ type II receptor (DNIIR) blocks TGFβ-mediated EMT in NMuMG cells (Fig 5A and B) and successfully prevents TGFβ-induced phosphorylation of Smad2 in the mouse 4T1 aggressive breast cancer tumor derived cell lines (Fig 5C and D) that represents an already well characterized model drastic reduction in lung metastasis occurring upon DNIIR expression (McEarchern et al, 2001; Ge et al, 2006; Padua et al, 2008; Liu et al, 2012). The 4T1 cells expressing WT or DN RII were injected into the MFP of NOD/SCID mice and effects on tumor growth, axonogenesis, and metastasis were analyzed. Whereas inhibition of TGFβ signaling (DN RII) had little impact on tumor growth, it suppressed the development of lung metastasis as previously described (McEarchern et al, 2001) (Fig 5E). Interestingly, DN RII overexpression also abolished tumor sympathetic-type innervation of the primary tumor (Fig 5F–H). Overall, our findings are clearly supportive of a role of primary tumor EMT program in axonogenesis and metastasis during breast cancer progression. Furthermore, we checked whether breast cancer progression is linked to primary tumor cell EMT program in human cancer by data mining the BRCA dataset from The Cancer Genome Atlas project (Fig 5I and J). We used tubulin β3 neuronal marker to rank human breast cancer primary tumors according to their nerve densities and showed a strong correlation with TGFβ1, fibronectin, N-cadherin, and Snail mesenchymal transcript expression as well as an inverse correlation with both ZO1 and catenin-β1 epithelial transcripts (Fig 5J). Finally, Kaplan–Meyer survival curve analysis validated the decrease in overall survival for patients with breast cancers having the highest levels of innervation as reflected by the expression of the neuronal marker TUBB3 (Fig 5K).

## Discussion

Platr18/LncENC1 was initially characterized for its functional integration in the transcriptional regulatory program of ESCs and more recently featured as one of several LncRNAs expressed during murine neuronal stem cell differentiation (Ivanova et al, 2006; Guttman et al, 2011; Bergmann et al, 2015; Sun et al, 2018; Carelli et al, 2019). Interestingly, we found that Platr18 is involved in primary tumor axonogenesis through the control of Sema4F, an axon guidance molecule involved in neural development (Armendáriz et al, 2012) and tumor innervation (Ayala et al, 2008; Ding et al, 2013) (Fig 2).

Beyond the specific context of the Platr18/Sema4F axis, we describe herein a novel role for TGFβ as regulator of primary tumor axonogenesis and link this new biological function to its well-described role as a regulator of later stages of tumor progression (Figs 3–5).

Despite being the most regulated LncRNA during E1KD-mediated or TGFβ-mediated EMT of the NMUMG cell model, Platr18 does not seems to control the EMT because its silencing is not sufficient to impairs TGFβ-mediated EMT and its overexpression does not trigger EMT (Fig S2). From our experimental observations, we established the link between TGFβ-mediated EMT and tumor axonogenesis. However, we did not observe a consensus about the molecular factors involved and no canonical axis was identified across different models. It will be of high interest in the future to detail the molecular mechanisms that are driving the EMT-mediated tumor axonogenesis in other biological contexts. Moreover, we did not observe TGFβ regulation of the Platr18/Sema4F axis in HepG2, 4T1, and MDA-MB-231, as we did in the PyMT-1099 and NMuMG models. Gene ontology analysis revealed even greater enrichment of nerve-related genes after silencing of hnRNP E1 or TGFβ treatment in these cells (Fig S4A and B). Furthermore, blocking TGFβ signaling in 4T1 cancer cells blocked tumor innervation (Fig 5). We therefore conclude that EMT program acts as a general trigger for axonogenesis and this is mediated through specific regulatory axes dependent on cellular and biological contexts. Because Sema4F silencing in NMuMG-E1KD cells and blockade of TGFβ signaling in 4T1 cells does not impact tumor growth in vivo, it constitutes appropriate and unbiased model to validate the pro-neurogenic role of TGFβ in primary breast tumors (Figs 4I and 5D). However, the reduced tumor outgrowth observed in vivo upon Platr18 silencing suggests that it has additional function(s) in tumor progression (Fig 4I). We did not observe significant impact of Sema4f silencing in tumor cell proliferation, primary tumor growth. However, Sema4F and other semaphorins also have major regulatory functions in cancer biology and it is also anticipated that the observed Sema4F up-regulation could also exert a tumor cell autonomous function, for instance by promoting neurite-like protrusions of tumor cells, or by influencing other tumor stroma cells, such as endothelial cells or cancer-associated fibroblasts (Tamagnone, 2012; Neufeld et al, 2016; Butti et al, 2018). For instance, Class3 semaphorins are well described for their functions in tumor growth and angiogenesis (Gaur et al, 2009). In a more general manner, semaphorins functions in the primary tumor biology are heavily dependent of the biological context because of their paracrine and autocrine activities (Neufeld et al, 2016). From the current study, we revealed the function of tumor cell plasticity in triggering tumor axonogenesis and the associated biological variables were differing across the biological models we used. Therefore, in the future, the elucidation of the exact molecular mechanisms, biological mediators and signaling pathways involved, dependent on the biological context, and the role of plasticity mediators beyond the TGFβ remain to be determined.

The function of axonogenesis in primary tumor progression and metastasis is an emerging area of investigation that may provide novel therapeutic opportunities. Recently, Mauffrey et al (2019) demonstrated in the context of prostate cancer that tumor infiltration by neural progenitors from the central nervous system drives tumor neurogenesis (Mauffrey et al, 2019). In breast cancer, Kamiya et al (2019) showed that increased sympathetic innervation promotes breast cancer progression and numerous studies have already correlated breast tumor innervation to metastasis and clinical outcomes (Zhao et al, 2014; Faulkner et al, 2019; Kamiya et al, 2019). In our biological context we detected only innervation of a sympathetic nature. Because we monitored EMT-driven late tumor progression in our models, such observation is consistent with the pro-tumorigenic role of sympathetic innervation (Magnon et al, 2013; Kamiya et al, 2019). Although sensory innervation, especially through nerve twigs could be associated with tumor progression (Madeo et al, 2018), we failed to detect nerves of a sensory nature. We also did not detect any parasympathetic innervation in our tumor models. This is consistent with previous observations that associate this nerve subtype to less aggressive tumors. It will be of interest to determine the exact nature of the sympathetic nerves that are infiltrated into the tumor. For instance, the β-adrenergic signaling have been identified for its critical role in the TME of many cancer types (Mravec et al, 2020) and both norepinephrine and epinephrine contribute to the initiation and progression of cancer (Barbieri et al, 2015; Bastos et al, 2018; Xia et al, 2019; Lamboy-Caraballo et al, 2020; Zhang et al, 2020), are associated to stress (Bastos et al, 2018; Zhang et al, 2019), and could be related to the EMT program (Shan et al, 2014). In other biological contexts, the β-adrenergic signaling have been observed as negatively influenced by the TGFβ cytokine (Iizuka et al, 1994; Nogami et al, 1994; Schluter et al, 1995; Huntgeburth et al, 2011). It will therefore be interesting to identify whether the β-adrenergic signaling is involved in the TGFβ induced tumor axonogenesis and to determine the function of other TME components such as fibroblast, smooth muscle, and endothelial cells. The characterization, identification, and understanding of the specific nature of tumor nerve components will require further investigations, and it will be interesting to associate specific biological contexts to specific innervation patterns. The link between tumor axonogenesis and EMT also appears specific of the biological context and the elucidation of associated mechanisms as well as the interplay between cancer cells, and the TME in this biological process still need further investigations.

The functional role of EMT during primary tumor axonogenesis may be related to the similarities between tumor related-EMT and neuroplasticity. Indeed, organization of neuronal circuits and tumor progression orchestrate common processes such as cell–cell contacts, cytoskeleton reorganization, and transcriptome reprogramming and thus share the expression of specific molecules such as neuronal cadherins or pluripotency transcription factors. We hypothesize that the expression of neuroplasticity-related factors activated during EMT may act as a decoy that mimic brain signals required for the homing of neural progenitors arising from the central nervous system.

Communication between the tumor and neuronal compartments could occur through direct contacts or secretion of proteins and/or exosomes (Madeo et al, 2018). However, the detailed mechanism(s) coordinating these exchanges and how they contribute to tumor progression still need further investigation (Vermeer, 2019).

Overall, by uncovering a new biological function of TGFβ on the tumor microenvironment, the present study may help reconcile some of the controversies regarding the precise role of EMT in tumor progression and metastasis.

## Materials and Methods

### Cell culture and reagents

NMuMG, 4T1, HEK293t, PC12, and Neuro2A cells were obtained from the American Type Culture Collection, and the MDA-MB-231-LM2 cells were graciously provided by Dr. Joan Massagué (Minn et al, 2005). The L1P and M1P cells that were used in the study are expressing a puromycin resistance gene that allows for their selection in vitro after isolation from the lungs. Cells were cultured in DMEM (Cat. No. SH30081.01; GE Healthcare Life Sciences) high glucose supplemented with 10% fetal bovine serum and 1% antibiotic/antimycotic solution (anti-anti–penicillin G, streptomycin, and amphotericin B). Primary cells and organoids culture were maintained in DMEM/F-12 supplemented with Anti–Anti, 10 mM Hepes (Cat. No. 15630-080; Gibco), 1× Glutamax (Cat. No. 35050-061; Gibco), Insulin 10 μg/ml (Sigma-Aldrich), B27 (1×; Gibco), EGF (50 ng.ml^−1^), FGF2 (5 ng.ml^−1^), Wnt3a (10 ng.ml^−1^), and R-Spondin-1 (50 ng.ml^−1^). All cells were cultured in a 37°C, 5% CO_2_ incubator. TGFβ was a generous gift from Genzyme Corporation. Antibody dilutions, company names, catalogue numbers and clone numbers, and their respective dilutions are listed below. Puromycin, blasticidin and G418 were purchased from InvivoGen.

### Spheroid culture

Female littermates at 6–9 wk of age were euthanized and the fourth inguinal MFP was isolated for mammary epithelial organoid culture. Fat pads were minced and treated with collagenase for 3 h. After dissociation, samples were centrifuged and resuspended in a 1:4 mixture of cold HF (Hanks’ Balanced Salt Solution supplemented with 2% FBS) and ammonium chloride solution. Samples were centrifuged at 350*g* for 5 min and pellets resuspended in DMEM/F12. Samples were centrifuged at 1,250*g* for 10 min, supernatant removed, and pellets resuspended in 10 ml DMEM/F12. Samples were pulsed to 1,250*g* for 3–4 s. Pulse centrifugation was repeated three more times after removal of supernatant and resuspension of pellet in 10 ml DMEM/F12.

Epithelial cell organoids were seeded in 50 μl Growth factor reduced Matrigel (Corning) at a density of 2 organoids/μl in 24 well plates. Pre-warmed organoid medium was added to wells following a 30–60 min incubation of cultures at 37°C to allow Matrigel to solidify.

### Lentivirus production

Lentiviral constructs targeting hnRNP E1, Platr18, Sema4F were obtained from the shRNA core at MUSC or cloned into the pLKO.1-neo construct (Addgene) using EcoR1/Age1 sites. HEK293t cells were grown to 60–70% confluence and transfected with the pLKO.1 shRNA plasmid containing the targeted hairpins, psPAX2, and pMD2.G packaging plasmids using Lipofectamine 3000 in OPTI-MEM. The medium was changed after overnight incubation to a fresh culture medium. Virus was collected and filtered at 24 and 48 h through a 0.45-μm sterile filter. For transduction, 1:5–1:2 ratios of virus containing media to culture media was incubated on the target cells with 8 μg/ml polybrene overnight.

### Care of animals and genetic models

All animal procedures are approved by the Animal Care and Use Committees of the Medical University of South Carolina. Mice carrying floxed alleles of PCBP1 on a C57BL/6 background were a kind gift from Dr. Philpott (NIDDK). Wild-type C57BL/6, Tg(MMTV-cre)4Mam/J mice, B6.FVB-Tg(MMTV-PyVT)634Mul/LellJ, FVB/N-Tg(MMTV-PyVT)634Mul/J, and FVB/NJ were purchased from the Jackson Laboratory.

PCBP1^fl/fl^ mice were crossed with the Tg(MMTV-cre)4Mam/J line to generate PCBP1^fl/fl^ MMTV-Cre^(+/−)^ and PCBP1^fl/fl^ MMTV-Cre^(−/−)^ mice. Triple mutant mice carrying PCBP1^fl/fl^ MMTV-Cre^(+/−)^ and PyVT^(+/−)^ were generated on a C57BL/6 background crossing with the B6.FVB-Tg(MMTV-PyVT)634Mul/LellJ line.

Male FVB/N-Tg(MMTV-PyVT)634Mul/J mice hemizygous for the PYVT transgene were crossed with noncarrier FVB/NJ females to generate PYVT females for experiments.

Primers used for genotyping are as follows: Cre-F: GCGGTCTGGCAGTAAAAACTATC and Cre-R GTGAAACAGCATTGCTGTCACTT. PYVT-F GGAAGCAAGTACTTCACAAGGG and PYVT-R GGAAAGTCACTAGGAGCAGGG. Primers used to detect the LoxP site upstream and downstream of PCBP1 were previously described (Ryu et al, 2017).

### Antibodies

Mouse monoclonal anti-E-cadherin (Clone [4A2], Cat. No. #14472, 1:2,000 dilution), Rabbit monoclonal anti-vimentin (Clone [D21H3], Cat. No. #5741, 1:2,000 dilution), Rabbit monoclonal anti-Smad2 (Clone [D43B4], Cat. No. #5339, 1:1,000 dilution), and Rabbit monoclonal anti-phospho-Smad2 (Ser465/467) (Clone [138D4], Cat. No. #3108, 1:1,000 dilution) were purchased from Cell Signaling Technology. Rabbit monoclonal anti-Ki67 (Clone [SP6], Cat. No. ab16667, 1:50 dilution) and rabbit polyclonal anti-tubulin-β3 (Cat. No. ab18207, 1:2,000 dilution) and polyclonal anti-ZO-1 (Cat. No. ab59720, 1:500 dilution) were purchased from Abcam, Inc. Mouse monoclonal anti-HSP90 (Clone [F-8], Cat. No. sc-13119, 1:10,000 dilution) was purchased from Santa Cruz Biotechnology, Inc. Rabbit polyclonal anti-tyrosine-hydroxylase (Cat. No. 25859-1-AP, 1:2,000 dilution) was purchased from Proteintech, Inc. Mouse monoclonal anti-hnRNP E1 (Clone [1G2] Cat. No. H00005093-M01, 1:1,000 dilution) was purchased from Abnova, Inc. Rat monoclonal anti-Sema4F (clone 780225 Cat. No. MAB7200, 1:200 dilution) was purchased from R&D Systems. Mouse monoclonal anti-Occludin (clone OC-3F10 Cat. No. 33–1500, 1:1,000 dilution) and Rabbit polyclonal anti-NGF (Cat. No. PA1-18377, 1:1,000 dilution) were purchased from Invitrogen. Mouse monoclonal anti–N-cadherin (clone 32 Cat. No. 610920, 1:1,000 dilution) was purchased from BD Transduction Laboratories.

### Primers

PCR primers were purchased from Eurofins Genomics:

Platr14-F GTGTAGGGGGACACCATCTG; Platr14-R TGAGCTGATTCCACTGAGACC; Platr16-F TGCCTCGTGGTAAGGAACTAC; Platr16-RCCTTTAACCTTCCACTGCTCT;
Platr18-F GCTCTGCTTCAGGGTTCCAT; Platr18-R TGGCAGGCCTTTGTGTAGAG; Platr20-F ATACGTGGAGGGAGTCACGA; Platr20-R GCGAGATTTGGCTCTTTGGC;
Vimentin-F AAGCACCCTGCAGTCATTCA; Vimentin-R TTGTACCATTCCTCGGCCTC;
E-cadherin-F GAAGGCTTGAGCACAACAGC; E-Cadherin-R AGATGGGGGCTTCATTCACG;
PCBP1-F CTGACTGGGCCTACCAATGC; PCBP1-R GCCGTACTGTTGGTCATGGA;
Sema4F-F AACGGTCAGCAGCTGTAATG; Sema4F-R AGCTCGGGAGATAATCGGCT;
FN1-FACGGTTTCCCATTACGCCAT; FN1-R TCATCCGCTGGCCATTTTCT;
TBP-F CGCAGCTTCAAAATATTGTATCTACC; TBP-R TCACTCTTGGCTCCTGTGC; GAPDH-F GGGTCCCAGCTTAGGTTCAT; GAPDH-R AGACACCAGTAGACTCCACG.

### Transfections

All cell transfections were carried out using 10 μg DNA per 10 ml of medium with cells at 70% confluence cultured in 100-mm plates. The transfection reagent Lipofectamine (Thermo Fisher Scientific) was used according to the protocol provided by the manufacturer.

### Western blot analysis

Western blot analysis was performed by standard SDS–PAGE. Whole cell lysates were prepared from 2 to 5 × 10^6^ cells in 300 μl of lysis buffer (20 mM Tris, pH 7.4, 1% Triton X-100, 10% glycerol, 137 mM NaCl, 2 mM EDTA, 1 mM Na_3_VO_4_, and protease inhibitors). Lysates were clarified by centrifugation at 4°C for 10 min in a Beckman tabletop microcentrifuge at maximum speed. Protein lysates were separated on 10 or 12% acrylamide minigels and transferred to immobilon-P membrane (Millipore). The membrane was blocked for 1 h in wash buffer (PBS containing 0.1% Tween 20) containing 5% nonfat dry milk followed by an overnight incubation with primary antibody diluted in the same blocking buffer. After extensive washing, the blot was incubated with secondary antibody for 1 h in blocking buffer, washed, and processed using the ECL+ Western blotting detection system (Amersham Biosciences).

### Immunofluorescence, FISH, and imaging

For immunofluorescence, cells were fixed for 15 min in PBS containing 3.7% (wt/vol) paraformaldehyde, followed by permeabilization with 0.2% (wt/vol) Triton X-100. Cells were then incubated 1 h in 3% BSA and incubated overnight with primary antibody in 2% BSA at 4°C. Then cells were incubated with secondary antibodies conjugated with Alexa Fluor (Life Technologies) at room temperature for 1 h followed by three washes with PBS before analysis with the FV10i confocal laser scanning microscope (Olympus).

### Single-molecule RNA FISH

Cells were fixed for 15 min in PBS containing 3.7% (wt/vol) paraformaldehyde, then slides were incubated overnight at 37°C in Stellaris hybridization solution containing the Platr18 probes set at 1:50 dilution. Cells are then washed three times in Stellaris wash buffers and stained with DAPI before imaging with Zeiss LSM 880 confocal microscope.

### Immunohistochemistry

Formalin-fixed, paraffin-embedded sections were deparaffinized in xylene, rehydrated in alcohol, and processed as follows. Sections were incubated with target retrieval solution (Dako) in a steamer for 45 min followed by 3% hydrogen peroxide solution for 10 min and protein block (Dako) for 20 min at room temperature. Sections were incubated overnight in a humid chamber at 4°C with antibody against Ki-67 (Clone [SP6], Cat. No. ab16667, 1:50 dilution) or against tubulin-β3 (Cat. No. ab18207, 1:2,000 dilution) purchased from Abcam Inc. followed by biotinylated secondary antibody (Vector laboratories) for 30 min and ABC reagent for 30 min. Immunocomplexes of horseradish peroxidase were visualized by DAB reaction (Dako), and sections were counterstained with hematoxylin before mounting. The tracking of the disseminated cells was performed by analysis of Ki67-positive cells into the lungs. The cancerous nature of the cells was validated by a certified pathologist.

### Fluorescence immunohistochemistry

Paraffin-embedded sections were deparaffinized in xylene, rehydrated in alcohol, and processed as follows. Sections were incubated with sodium citrate 10 mM retrieval solution in a steamer for 20 min followed by 10 min in deionized water and 30 min in blocking buffer (1% bovine calf serum in 1× phosphate buffer saline) at room temperature. Sections were incubated overnight in a humid chamber at 4°C with antibody against tubulin-β3 (Cat. No. Ab78078, 1:1,000 dilution) and tyrosine hydroxylase (Cat. No. ab75875, 1:100 dilution) purchased from Abcam and acetylcholine transferase (Cat. No. ab178850, 1:500 dilution) purchased from Abcam or TRPV1 (Cat. No. ACC-030, 1:500 dilution) purchased from Alomone labs, followed by fluorescent secondary antibodies Alexa Fluor 488/568–conjugated secondary antibodies (Cat. No. A11029 & A11036 1:11,000 dilution; purchased from Invitrogen) for 1 h at room temperature and then 5 min in Hoechst 33342 (Cat. No. 62249 1:1,000 dilution) before mounting. Fluorescent immunocomplexes were visualized by confocal microscopy.

### PCR analysis

Total RNA was isolated using TRIzol (Thermo Fisher Scientific). cDNA synthesis was performed using qScript cDNA synthesis kits with 100–1,000 ng of total RNA (Quantabio). Semi-quantitative PCR was conducted using Maxima Hot Start PCR Master Mix (Thermo Fisher Scientific). Real-time quantitative PCR was conducted using iQ SYBR Green Supermix (Bio-Rad) using CFX384 Real-Time System (Bio-Rad). Relative gene expression was calculated using RFX Manager software, and genes were normalized to TBP internal control.

### RNA/DNA sequencing

RNA sequencing was performed using the MUSC genomics shared resource. RNA was extracted by Trizol/phenol chloroform and analyzed for quality (RIN) by a Bioanalyzer (Agilent 2100). RNA was considered adequate for sequencing with RIN 7. After library preparation, single-pass sequencing runs were performed on an Illumina HiSeq2500 instrument. Read quality was assessed with FastQC. For RNA sequencing, the reads were aligned to M18 mouse genome assembly or GRCh38 human genome assembly with Bowtie2 and quantification to references was carried out with Partek (E/M). Differential expression analysis was performed in Partek Flow using the Gene Specific Analysis algorithm.

## Data Availability Statement

The data that support the findings of this study are available from the corresponding authors upon reasonable request. Bioinformatic data are available under the Gene Expression Omnibus accession numbers: GSE112797, GSE94637, GSE114572, GSE110912, GSE112797 GSE94637, and GSE146273.

### Statistical analyses

All statistical analyses were performed using Prism 7 (Graphpad) using one-way ANOVA analysis and *t* test. Human tumor samples analysis from The Cancer Genome Atlas were normalized in Log_2_ (Normalized count + 1) and subjected to Pearson rho scores and *P*-values analysis using the UCSC Xena platform. No statistical method was used to predetermine sample size and experiments were not randomized, and we were not blinded to allocation during experiments and outcome assessment. All the results are expressed as mean ± SD or other representations are specified in the figure legends. *P < 0.05; **P < 0.01; ***P < 0.001.

## Supplementary Material

Reviewer comments
