# Supplementary material for: TGFβ-induced expression of long noncoding lincRNA Platr18 controls breast cancer axonogenesis
Source: Life Sci Alliance. 2021 Nov 22;5(2):e202101261. doi: 10.26508/lsa.202101261 (PMC8645334; doi:10.26508/lsa.202101261)
Supplement: Reviewer comments [file LSA-2021-01261_review_history.pdf]

TGFβ-induced expression of long non-coding lincRNA Platr18 controls breast cancer axonogenesis

Cécile Fréreux, Clémence Obéllianne, Ken Noguchi, Breege V Howley, Annamarie C Dalton, Philip H Howe and Simon Grelet
DOI: 10.26508/lsa.202101261

Corresponding author(s): Dr. Simon Grelet (University of South Alabama)

Review Timeline:

Submission Date:	2021-10-15
Editorial Decision:	2021-10-18
Revision Received:	2021-10-19
Editorial Decision:	2021-10-21
Revision Received:	2021-10-29
Accepted:	2021-11-01

Scientific Editor: Dr. Eric Sawey, PhD

Transaction Report:
(Note: With the exception of the correction of typographical or spelling errorsthat could be a source of ambiguity, letters and reports are not edited. The original formatting of letters and referee reports may not be reflected in this compilation.)

Please note that the manuscript was previously reviewed at another journal and the reports were taken into account in the decision-making process at *Life Science Alliance*.

No full Peer Review Process File is available with this article, as the authors have chosen not to make the review process public in this case.

1st Editorial Decision October 18, 2021

October 18, 2021

Re: Life Science Alliance manuscript #LSA-2021-01261-T

Dr. Simon Grelet
University of South Alabama
Biochemistry and Molecular Biology
Mitchell Cancer Institute 1660 Spring Hill AveMobile, AL 36604

Dear Dr. Grelet,

Thank you for submitting your manuscript entitled "TGFβ-induced expression of pluripotency associated LincRNA Platr18 controls breast cancer axonogenesis and progression" to Life Science Alliance. We inviteyou to submit a revised manuscript addressing Reviewer 1's remaining concerns via Discussion.

To upload the revised version of your manuscript, please log in to youraccount: https://lsa.msubmit.net/cgi-bin/main.plex

You will be guided to complete the submission of your revised manuscriptand to fill in all necessary information. Please get in touch in case you do not know or remember your login name.

While you are revising your manuscript, please also attend to the below editorial points to help expedite the publication of your manuscript. Pleasedirect any editorial questions to the journal office.

The typical timeframe for revisions is three months. Please note that papersare generally considered through only one revision cycle, so strong supportfrom the referees on the revised version is needed for acceptance.

When submitting the revision, please include a letter addressing thereviewers' comments point by point.

Thank you for this interesting contribution to Life Science Alliance. We arelooking forward to receiving your revised manuscript.

Sincerely,

Eric Sawey, PhD
Executive Editor
Life Science Alliance
http://www.lsajournal.org

A. THESE ITEMS ARE REQUIRED FOR REVISIONS

-- A letter addressing the reviewers' comments point by point.

-- An editable version of the final text (.DOC or .DOCX) is needed forcopyediting (no PDFs).

-- High-resolution figure, supplementary figure and video files uploaded asindividual files: See our detailed guidelines for preparing your production- ready images, https://www.life-science-alliance.org/authors

-- Summary blurb (enter in submission system): A short text summarizing ina single sentence the study (max. 200 characters including spaces). This text is used in conjunction with the titles of papers, hence should be informative and complementary to the title and running title. It should describe the context and significance of the findings for a general readership; it should be written in the present tense and refer to the work inthe third person. Author names should not be mentioned.

-- By submitting a revision, you attest that you are aware of our payment policies found here: https://www.life-science-alliance.org/copyright-license-fee

B. MANUSCRIPT ORGANIZATION AND FORMATTING:

Full guidelines are available on our Instructions for Authors page,https://www.life-science-alliance.org/authors

We encourage our authors to provide original source data, particularly uncropped/-processed electrophoretic blots and spreadsheets for the mainfigures of the manuscript. If you would like to add source data, we would welcome one PDF/Excel-file per figure for this information. These files will be linked online as supplementary "Source Data" files.

IMPORTANT: It is Life Science Alliance policy that if requested, original data images must be made available. Failure to provide original images upon request will result in unavoidable delays in publication. Please ensurethat you have access to all original microscopy and blot data images beforesubmitting your revision.

1st Revision - Editorial Decision October 21, 2021

October 21, 2021

RE: Life Science Alliance Manuscript #LSA-2021-01261-TR

Dr. Simon Grelet
University of South Alabama
Biochemistry and Molecular Biology
Mitchell Cancer Institute 1660 Spring Hill AveMobile, AL 36604

Dear Dr. Grelet,

Thank you for submitting your revised manuscript entitled "TGFβ-induced LincRNA Platr18 controls breast cancer axonogenesis and progression". We would be happy to publish your paper in Life Science Alliance pending final revisions necessary to meet our formatting guidelines.

Along with points mentioned below, please tend to the following:

-Please upload all figure files as individual ones, including thesupplementary figure files
-please add the Twitter handle of your host institute/organization as well asyour own or/and one of the authors in our system
-please note that titles in the system and manuscript file must match
-please use the [10 author names, et al.] format in your references (i.e. limitthe author names to the first 10)
-please add your main, supplementary figure, and table legends to the mainmanuscript text after the references section
-we encourage you to revise the figure legend for figure 5 such that thefigure panels are introduced in alphabetical order
-there is a callout for figure S5 in the manuscript text (please correct)
-please upload your Tables in editable .doc or excel format
-please indicate molecular weights next to each protein blot
-please add scale bars to microscopy images, and indicate the size in thecorresponding figure legends

If you are planning a press release on your work, please inform us immediately to allow informing our production team and scheduling arelease date.

LSA now encourages authors to provide a 30-60 second video where the study is briefly explained. We will use these videos on social media to promote the published paper and the presenting author (for examples, see https://twitter.com/LSAjournal/timelines/1437405065917124608).
Corresponding or first-authors are welcome to submit the video. Pleasesubmit only one video per manuscript. The video can be emailed to contact@life-science-alliance.org

To upload the final version of your manuscript, please log in to youraccount:
https://lsa.msubmit.net/cgi-bin/main.plex

You will be guided to complete the submission of your revised manuscriptand to fill in all necessary information. Please get in touch in case you do not know or remember your login name.

To avoid unnecessary delays in the acceptance and publication of yourpaper, please read the following information carefully.

A. FINAL FILES:

These items are required for acceptance.

-- An editable version of the final text (.DOC or .DOCX) is needed forcopyediting (no PDFs).

-- High-resolution figure, supplementary figure and video files uploaded asindividual files: See our detailed guidelines for preparing your production- ready images, https://www.life-science-alliance.org/authors

-- Summary blurb (enter in submission system): A short text summarizing ina single sentence the study (max. 200 characters including spaces). This text is used in conjunction with the titles of papers, hence should be informative and complementary to the title. It should describe the context and significance of the findings for a general readership; it should be writtenin the present tense and refer to the work in the third person. Author namesshould not be mentioned.

B. MANUSCRIPT ORGANIZATION AND FORMATTING:

Full guidelines are available on our Instructions for Authors page,https://www.life-science-alliance.org/authors

We encourage our authors to provide original source data, particularly uncropped/-processed electrophoretic blots and spreadsheets for the mainfigures of the manuscript. If you would like to add source data, we would welcome one PDF/Excel-file per figure for this information. These files will be linked online as supplementary "Source Data" files.

Submission of a paper that does not conform to Life Science Allianceguidelines will delay the acceptance of your manuscript.

It is Life Science Alliance policy that if requested, original data images must be made available to the editors. Failure to provide original images upon request will result in unavoidable delays in publication. Please ensurethat you have access to all original data images prior to final submission.

The license to publish form must be signed before your manuscript can besent to production. A link to the electronic license to publish form will be sent to the corresponding author only. Please take a moment to check your funder requirements.

Reviews, decision letters, and point-by-point responses associated with peer-review at Life Science Alliance will be published online, alongside themanuscript. If you do want to opt out of having the reviewer reports and your point-by-point responses displayed, please let us know immediately.

Thank you for your attention to these final processing requirements. Pleaserevise and format the manuscript and upload materials within 7 days.

Thank you for this interesting contribution, we look forward to publishingyour paper in Life Science Alliance.

Sincerely,

Eric Sawey, PhD
Executive Editor
Life Science Alliance
http://www.lsajournal.org

2nd Revision - Editorial Decision November 1, 2021

November 1, 2021

RE: Life Science Alliance Manuscript #LSA-2021-01261-TRR

Dr. Simon Grelet
University of South Alabama
Biochemistry and Molecular Biology
Mitchell Cancer Institute 1660 Spring Hill Ave
Mobile, AL 36604

Dear Dr. Grelet,

Thank you for submitting your Research Article entitled "TGFβ-induced expression of long non-coding lincRNA Platr18 controls breast cancer axonogenesis". It is a pleasure to let you know that your manuscript is now accepted for publication in Life Science Alliance. Congratulations on this interesting work.

The final published version of your manuscript will be deposited by us to PubMed Central upon online publication.

Your manuscript will now progress through copyediting and proofing. It is journal policy that authors provide original data upon request.

Reviews, decision letters, and point-by-point responses associated with peer-review at Life Science Alliance will be published online, alongside the manuscript. If you do want to opt out of having the reviewer reports and your point-by-point responses displayed, please let us know immediately.

IMPORTANT: If you will be unreachable at any time, please provide us with the email address of an alternate author. Failure to respond to routine queries may lead to unavoidable delays in publication.

Scheduling details will be available from our production department. You will receive proofs shortly before the publication date. Only essential corrections can be made at the proof stage so if there are any minor final changes you wish to make to the manuscript, please let the journal office know now.

DISTRIBUTION OF MATERIALS:
Authors are required to distribute freely any materials used in experiments published in Life Science Alliance. Authors are encouraged to deposit materials used in their studies to the appropriate repositories for distribution to researchers.

You can contact the journal office with any questions, contact@life-science-alliance.org

Again, congratulations on a very nice paper. I hope you found the review process to be constructive and are pleased with how the manuscript was handled editorially. We look forward to future exciting submissions from your lab.

Sincerely,

Eric Sawey, PhD
Executive Editor
Life Science Alliance
http://www.lsajournal.org
